# NPM1 Mutational Status Underlines Different Biological Features in Pediatric AML

**DOI:** 10.3390/cancers13143457

**Published:** 2021-07-10

**Authors:** Claudia Tregnago, Maddalena Benetton, Davide Padrin, Katia Polato, Giulia Borella, Ambra Da Ros, Anna Marchetti, Elena Porcù, Francesca Del Bufalo, Cristina Mecucci, Franco Locatelli, Martina Pigazzi

**Affiliations:** 1 Department of Women’s and Children’s Health, Haematology-Oncology Clinic and Lab, University of Padova, 35128 Padova, Italy; claudia.tregnago@unipd.it (C.T.); maddalena.benetton@studenti.unipd.it (M.B.); davide.padrin@aopd.veneto.it (D.P.); katia.polato@unipd.it (K.P.); giulia.borella@unipd.it (G.B.); ambra.daros@studenti.unipd.it (A.D.R.); anna.marchetti.4@studenti.unipd.it (A.M.); elena.porcu@unipd.it (E.P.); 2 Department of Pediatric Hematology and Oncology, IRCCS Bambino Gesù Children’s Hospital, 00165 Roma, Italy; francesca.delbufalo@opbg.net (F.D.B.); franco.locatelli@opbg.net (F.L.); 3 Division of Hematology and Clinical Immunology, Department of Medicine, University of Perugia, 06123 Perugia, Italy; cristina.mecucci@unipg.it; 4 Department of Pediatrics, Sapienza University of Rome, 00185 Roma, Italy

**Keywords:** Nucleophosmin, NPM1, acute myeloid leukemia, gene expression, TP53, mutation, genetic, HOX genes, drug treatment

## Abstract

**Simple Summary:**

Nucleophosmin (NPM1) protein regulates several cellular processes and is predominantly located in the nucleolus, owing to the localization signal provided by two tryptophan residues. In acute myeloid leukemia (AML), NPM1 gene is frequently mutated, leading to the aberrant translocation of the protein into cytoplasm. In the present work, we classified NPM1 mutations according to the loss of either one or both tryptophan residues as non-A-like and A-like mutations, respectively, and evaluated their biological features. We found that non-A-like mutations partially delocalize NPM1 protein into the cytoplasm, with a proportion of remaining nucleolar protein preserving p53 protein expression and downstream activity. Different HOXA and HOXB gene expression and cell death pathway activation between A-like and non-A-like NPM1-mutated cells were shown, with an enhanced sensitivity to chemotherapy for AML cells with non-A-like mutations. This study suggests the need for a sub-classification of NPM1-mutated AML, with subsequent implications in the therapeutic management.

**Abstract:**

Nucleophosmin (NPM1) is a nucleocytoplasmic shuttling protein, predominantly located in the nucleolus, that regulates a multiplicity of different biological processes. NPM1 localization in the cell is finely tuned by specific signal motifs, with two tryptophan residues (Trp) being essential for the nucleolar localization. In acute myeloid leukemia (AML), several NPM1 mutations have been reported, all resulting in cytoplasmic delocalization, but the putative biological and clinical significance of different variants are still debated. We explored HOXA and HOXB gene expression profile in AML patients and found a differential expression between NPM1 mutations inducing the loss of two (A-like) Trp residues and those determining the loss of one Trp residue (non-A-like). We thus expressed NPM1 A-like- or non-A-like-mutated vectors in AML cell lines finding that NPM1 partially remained in the nucleolus in the non-A-like NPM1-mutated cells. As a result, only in A-like-mutated cells we detected HOXA5, HOXA10, and HOXB5 hyper-expression and p14ARF/p21/p53 pathway deregulation, leading to reduced sensitivity to the treatment with either chemotherapy or Venetoclax, as compared to non-A-like cells. Overall, we identified that the NPM1 mutational status mediates crucial biological characteristics of AML cells, providing the basis for further sub-classification and, potentially, management of this subgroup of patients.

## 1. Introduction

Nucleophosmin (NPM1) is a multifunctional, nucleocytoplasmic shuttling protein, that shows a predominant nucleolar localization [1,2]. It performs several biological functions in different cellular compartments, including: molecular chaperoning, ribosome biogenesis, DNA repair, preservation of genomic stability, and regulation of apoptosis [3,4] (Supplemental Figure S1) by controlling the activity of several partner proteins [5]. For instance, NPM1 allows the correct localization of p14ARF in the nucleolus: in presence of an oncogenic stress, it triggers the activation of the p14ARF-p53 pro-apoptotic pathway, promoting MDM2 degradation, which, in turn, increases the stability of p53 and the levels of its downstream targets, such as p21. These mechanisms converge in regulating apoptosis [5]. However, when NPM1 is located in the cytoplasm, it exerts anti-apoptotic properties through the binding and inhibition of the activated forms of caspase-6 and -8, antagonizing the caspase-dependent apoptosis [6]. Furthermore, the overexpression of NPM1 prevents the mitochondrial translocation of p53, necessary for the release of cytochrome C and the initiation of the intrinsic pathway of apoptosis [7].

The nucleus-cytoplasm shuttling of NPM1 is regulated by specific signal motifs. A bipartite nuclear localization signal (NLS) drives NPM1 from the cytoplasm to the nucleoplasm. NPM1 then localizes in the nucleolus through an aromatic nucleolar localization signal (NoLS), a 3-helix structure located at the C-terminus. NoLS contains two tryptophan residues (W288 and W290) that stabilize the hydrophobic core of triple helix, enabling its proper folding [8,9,10,11].

The human NPM1 gene is located on chromosome 5q35 and contains 12 exons. NPM1 mutations have been identified in about one-third of adults and 6–8% of pediatric patients with acute myeloid leukemia (AML) [12,13,14,15], accounting for a distinct entity [16]. In pediatric AML, NPM1 mutations are associated with peculiar biological and clinical features [10,13,17], conferring an independent favorable prognostic impact when occurring as isolated aberrancy [18]. Moreover, NPM1-mutated AML blasts have a peculiar gene expression profile, characterized by low levels of CD34 and hyper-expression of several of the HOX family genes [19].

NPM1 mutations significantly associate with normal karyotype and often with abnormalities of the FLT3 gene, in particular with the internal tandem duplication (ITD). The favorable prognostic impact in the FLT3ITD mutation for pediatric AML is still controversial [12,18].

To date, more than 40 NPM1 variants have been reported in adult AML [20,21]. Most of them are located in the C-terminal region, causing the loss of the NoLS and the consequent aberrant localization of the protein to the cytoplasm [10,22,23]. The most common mutation, namely type A, accounts for ~80% of all variants in adults AML and for 11–50% of all mutations in pediatric AML [24], indicating that the type of NPM1 alterations are different between adult and pediatric cases. The biological and clinical significance of these variants is still debated, with some research groups reporting a correlation between the different NPM1 mutations and outcome, while others did not observe any prognostic impact [20,25,26]. Considering the intrinsic correlation between NPM1 localization and function, one important distinction must be considered between the loss of both Trp residues of the NoLS (occurring in the 95% of variants), and the deletion of only one residue (5%) [27].

Here, we aimed to investigate the heterogeneity of NPM1 mutations, and found that NPM1 partially remains in the nucleolus when one Trp residue within NoLS is maintained. This diversity translates into distinct HOX gene expression and p14ARF-p53 pro-apoptotic pathway activation, with a different susceptibility to drug treatment.

## 2. Materials and Methods

Vector construction and site-specific mutagenesis. pEGFP-C1-NPM1wt vector was previously described [21]; NPM1 was cloned into the multiple cloning site (MCS), in frame with enhanced green fluorescent protein (EGFP). We performed site-specific mutagenesis, following the protocol described by Liu et.al. [28], to obtain different A-like NPM1 mutations (namely A, B and type D), and other non-A-like mutations (namely type 5 and 7). The primers used for site-specific mutagenesis are listed in Table 1.

In vitro cell culture and treatments. The leukemia cell line HL-60 (DMSZ, Braunschweig, Germany) was maintained in RPMI 1640 (Thermo Fisher Scientific, Waltham, MA, USA), while SHI-1 (DMSZ) in DMEM (Thermo Fisher Scientific). All the media were supplemented with 10% FBS (Thermo Fisher Scientific), 2 mM glutamine (Gibco, Life Technologies, Carlsbad, CA, USA), and 100 U/mL streptomycin/penicillin (Gibco, Life Technologies). Primary cells were obtained by bone marrow samples of pediatric patients affected by de novo AML, provided by the pediatric OncoHematology Lab of Padova Hospital, being the Associazione Italiana Emato Oncologia Pediatrica (AIEOP) reference center for the pediatric AML diagnosis. Diagnosis of leukemia was established according to standard criteria based on immune-histochemical staining, immune-phenotyping, cytogenetic studies, and molecular genetics, as detailed in the AIEOP AML 2002/01 treatment protocol [14]. Primary cells were cultured in RPMI Medium 1640 with 10% FBS, 2 mM glutamine and 100 U/mL streptomycin/penicillin, supplemented with cytokines (50 ng/mL hTPO, 50 ng/mL hSCF, 50 ng/mL hFlt3L, 20 ng/mL hIL-3 and 20 ng/mL hIL-6, Miltenyi Biotec, Bergisch Gladbach, Germany).

Cell transfection. Transient cell transfection was performed using the Nucleofector technology (Amaxa Biosystems, Lonza, Basel, Switzerland), according to the manufacturer’s instructions. Briefly, 5 × 10^6^ HL-60 or SHI-1 were nucleofected with 1.2 μg of pEGFP plasmids, using Sol V (Lonza).

Immunofluorescence. EGFP fluorescent cells were analyzed after cytospin, 8 h post transfection, using Zeiss 800 Confocal microscope (Zeiss, Oberkochen, Germany). Nuclei were stained with DAPI (Sigma Aldrich-Merck Millipore, Darmstadt, Germany).

Western Blot. Western blots were hybridized with anti-GFP (B-2, sc-9996), anti-p53 (Pab 1801), anti-HDAC1 (H-51, sc-7872), anti-GAPDH (0411, sc-47724), all from Santa Cruz Biotecnology (Santa Cruz, CA, USA); anti-p21 (ab109199, Abcam, Cambridge, UK), anti-p14ARF (MA5-14260, Thermo Fisher Scientific). The horseradish peroxidase-conjugated secondary antibody was either anti-rabbit or mouse (Merck Millipore). Immunoblotting was performed as previously described [29], and signal was quantified using ImageJ software. Nuclear protein extraction was performed with the Subcellular Protein Fractionation Kit for Cultured Cells (78840, Thermo Fisher Scientific), following the manufacturer’s instructions.

Gene expression (GEP) analysis. Gene expression data were downloaded from GEO Database (GSE75461) [30]. Unsupervised clusterings were created using the ‘heatmap.plus’ function from the heatmap.plus’ R package with the ‘canberra’ as distance method and ‘ward.D2′ as the clustering algorithm and visualized using the ‘heatmap3′ function from the ‘hetamap3′ package. Dotplots were produced using ‘Graphics’ R package.

Quantitative real-time PCR. Total RNA was isolated using Trizol (Invitrogen—Thermo Fisher Scientific). One μg of RNA was reverse-transcribed into cDNA using the SuperScript II system (Invitrogen—Thermo Fisher Scientific) according to the manufacturer’s instructions. Expression of mRNA was measured by real time PCR (RQ-PCR) on an ABI 7900HD platform (Applied Biosystems, Foster City, CA, USA) using the Platinum™ SYBR™ Green qPCR SuperMix (Invitrogen—Thermo Fisher Scientific) and normalized on GUS housekeeping gene, using the 2^−ΔΔCt^ method.

Apoptosis analysis. Apoptosis was evaluated by double staining with Annexin-V/7-Aminoactinomycin D (7AAD, BD Biosciences, Franklin Lakes, NJ, USA) and analyzed using Cytomic FC500 (Beckman Coulter, Brea, CA, USA). Apoptotic cells were expressed as the percentage of Annexin-V-positive and/or 7AAD-positive cells and were compared to control (DMSO).

Cell viability assay. Cell viability of AML cells was evaluated using CellTiter-Glo^®^ assay (Promega Fitchburg, WI, USA) following the manufacturer’s guidelines. Briefly, 50 × 10^3^ cells in 100 μL of proper medium were cultured in sterile 96-well plates and incubated at 37 °C. Cell viability-ATP production was evaluated at the indicated time points by adding 100 μL CellTiter-Glo^®^ reagent, shaking plates for 2 min then incubating for 20 min at room temperature and recording the luminescence with the VICTOR3TM multilabel plate reader (Perikin Elmer, Waltham, MA, USA) with an integration time of 1 s per well.

Data evaluation and statistical analyses. The *t*-test was adopted for significance between differences in means when two groups were evaluated; ANOVA was used to compare more than two groups. Graphs and associated statistical analyses were generated using GraphPad Prism 8 (GraphPad, La Jolla, CA, USA). All data are presented as mean ± standard error of the mean (S.E.M.). * *p*-value < 0.05, ** *p*-value < 0.01, *** *p*-value < 0.001, **** *p*-value < 0.0001 were considered statistically significant.

## 3. Results

### 3.1. Intracellular Localization of the EGFP-NPM1 Mutants

**Figure 1:**
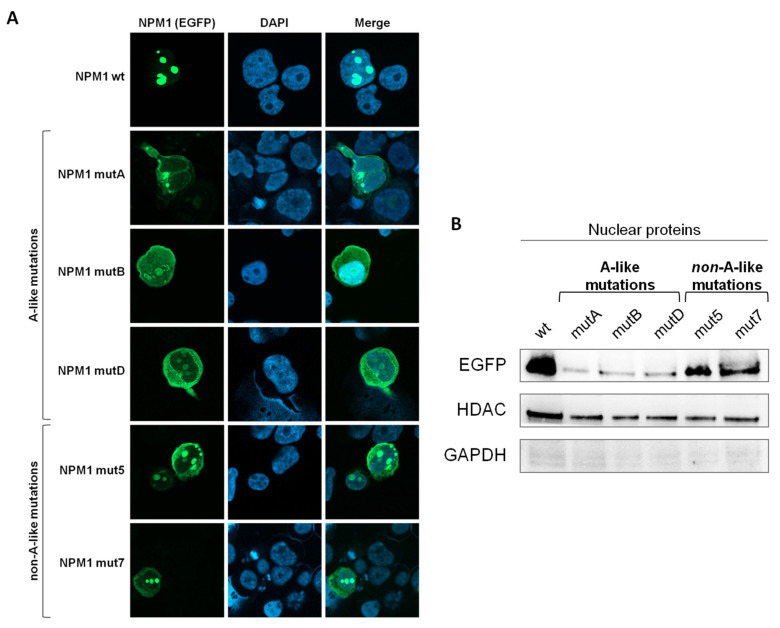
Intracellular localization of mutated NPM1. (**A**) Immunofluorescence of HL-60 AML cells transfected with pEGFP-NPM1wt, pEGFP-NPM1mutA, pEGFP-NPM1mutB, pEGFP-NPM1mutD, pEGFP-NPM1mut5, and pEGFP-NPM1mut7 plasmids, where EFGP is fused to NPM1. EGFP signal (green) represents NPM1 protein, and nuclei are stained with DAPI (blue) (40× magnification, scale bar = 15 μm). (**B**) Western blot showing EGFP protein on nuclear fraction of EGFP positive HL-60 cells transfected with pEGFP-NPM1wt, pEGFP-NPM1mutA, pEGFP-NPM1mutB, pEGFP-NPM1mutD, pEGFP-NPM1mut5, and pEGFP-NPM1mut7 plasmids. The whole western blot images are in Figure S5.

In order to evaluate the intracellular localization of mutated NPM1, we generated EGFP-NPM1mut constructs and transfected HL-60 cells. Briefly, by mutagenesis of the NPM1 wild type plasmid [21] we generated vectors expressing NPM1 mutation type A, B and NPM1 mutation D with the loss of both Trp residues (A-like mutations) and vectors expressing NPM1 mutation 7 and 8, where only one Trp residue is lost (non-A-like mutations) derived from de novo NPM1-mutated AML samples. NPM1 protein localization was then studied with both fluorescence microscopy and Western blot after nucleus-cytoplasmic protein fractionation. We confirmed that NPM1wt was physiologically located into nucleolous and that NPM1mutA, NPM1mutB, and NPM1mutD were predominantly expressed in the cytoplasm. We appreciate that the NPM1mut5 and NPM1mut7 were found co-expressed in both nucleolus and cytoplasm (Figure 1A). To support the intracellular localization differences between NPM1 mutational subtypes, HL-60 cells were transfected with either the pEGFP-NPM1wt vector, or A-like pEGFP-NPM1mutA, B, D vectors or the non-A-like pEGFP-NPM1mut5, 7 vectors. EGFP+ sorted cells showed that the NPM1 protein was detected in the nuclear fraction of NPM1wt and of the non-A-like NPM1-transfected cells, whereas it was severely reduced in the nuclear compartment of the A-like NPM1-transfected cells (Figure 1B). This latter finding confirmed that A-like mutations cause the loss of the nucleolar localization of NPM1 protein, whereas in non-A-like mutations NPM1 partially remained in the nucleolus, suggesting it may rescue a residual physiological functionality.

### 3.2. Effects of Different NPM1 Mutations on HOX Genes Expression

**Figure 2:**
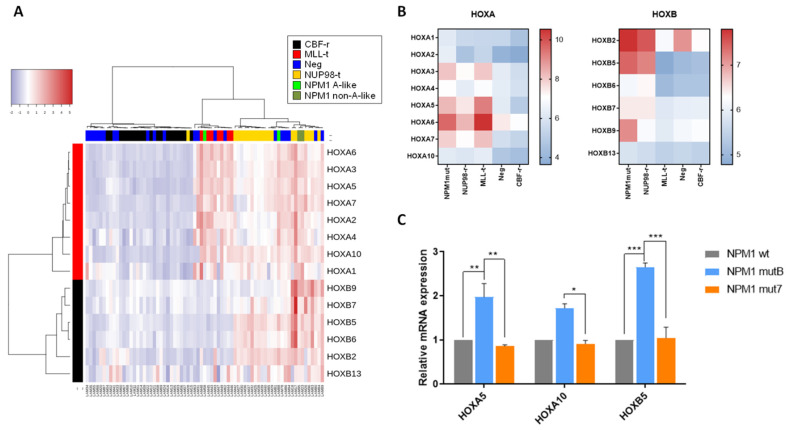
HOXA and HOXB gene expression according to NPM1 mutations. (**A**) Hierarchical clustering analyses of 71 AML patients for HOXA and HOXB family genes expression. AML genetic subtypes are reported in the legend with color codes. (**B**) Heatmap visualization of the median expression of HOXA and HOXB genes within the different AML genetic subtypes: NPM1 mutations n = 4, MLL translocations n = 7, NUP98 translocations n = 19, negative for tested molecular markers n = 21, CBF rearrangements n = 20. (**C**) mRNA expression levels of HOXA5, HOXA10 and HOXB5 in HL-60 cells transfected with pEGFP-NPM1wt, pEGFP-NPM1mutB (A-like), and pEGFP-NPM1mut7 (non-A-like), evaluated by RQ-PCR. Data are presented as mean of three independent experiments ± SEM. * *p*-value < 0.05, ** *p*-value < 0.01, *** *p*-value < 0.001.

Aberrant expression of HOX genes has been frequently associated with specific AML genetic subtypes, including NPM1-mutated. Therefore, we next evaluated HOX gene expression, in order to identify a possible correlation with the different NPM1 mutations. Through the analysis of our previously described pediatric AML cohort gene expression signature (n = 71, GSE75461 [30]: NPM1 mutations n = 4, MLL-translocations n = 7, NUP98-rearrangements n = 19, no molecular marker n = 21, CBF rearrangements n = 20), we documented that HOXA family genes were overexpressed in NPM1-mutated cells, as well as in MLL-translocated and NUP98-rearranged AML blasts. The unsupervised analysis revealed that NPM1-mutated patients cluster together with MLL-translocated and NUP98-rearranged AML, but separately from the risk-matched CBF-rearranged AML (Figure 2A). HOXA family genes resulted highly expressed in MLL-translocated, NUP98-rearranged, and NPM1-mutated AML as expected, whereas HOXB genes were mostly upregulated in NUP98-rearranged and NPM1-mutated AML (Figure 2B, Supplemental Figure S2). We then analyzed the gene expression data according to the NPM1 mutational type, finding that A-like NPM1-mutated AML cases show a generally higher expression of the HOXA genes compared to non-A-like NPM1-mutated ones (Supplemental Figure S3). Of note, A-like and non-A-like NPM1-mutated patients were distributed in separate dendrogram branches (green boxes in Figure 2A). To obtain deeper insights into the correlation between HOX family gene expression and NPM1 mutational status, we transfected HL-60 with the pEGFP-NPM1wt, pEGFP-NPM1mutB, and pEGFP-NPM1mut7 plasmids and analyzed HOXA5, HOXA10, and HOXB5 expression in EGFP+-sorted cells, previously described as the most aberrantly expressed HOX genes in NPM1-mutated AML [31]. We confirmed that these genes were significantly increased when A-like NPM1 mutation (mutB) vector was expressed as compared to the non-A-like (mut7, Figure 2C; * *p* < 0.05, ** *p* < 0.01, *** *p* < 0.001), supporting the hypothesis that the activation of crucial HOX genes differ according to the mutation type.

### 3.3. NPM1 Mutational Status and p53

**Figure 3:**
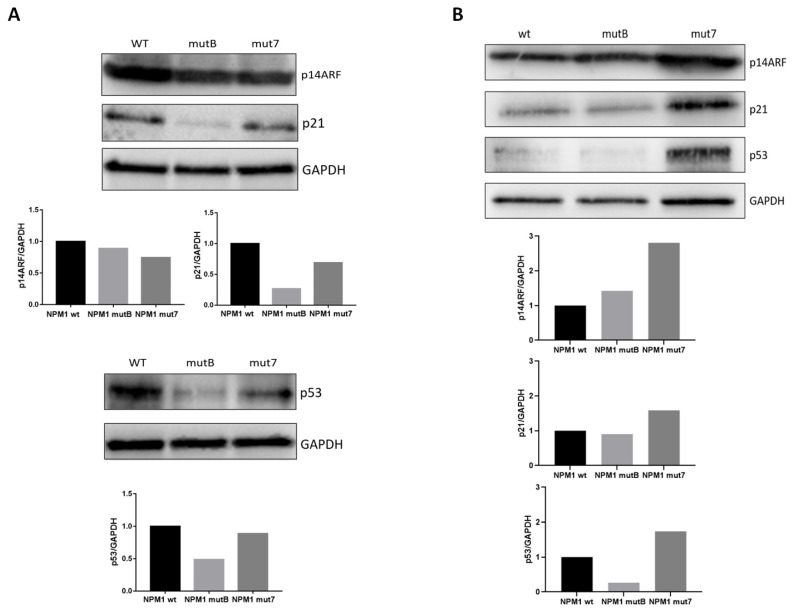
Impact of different NPM1 mutations on p53 pathway. (**A**,**B**) Western blot showing p14ARF, p21, and p53 protein levels in HL-60 (**A**) and SHI-1 cells (**B**) transfected with pEGFP-NPM1wt, pEGFP-NPM1mutB (A-like), and pEGFP-NPM1mut7 (non-A-like) plasmids. GAPDH was used as calibrator. Histograms show protein quantification using ImageJ software, and normalized with respect to GAPDH levels.

We hypothesized that different NPM1 mutations could affect the physiological nucleolar localization of one of its main targets, p14ARF and, consequently, its function, inducing aberrant p14ARF/p53/p21 pro-apoptotic signaling. We therefore analyzed p14ARF/p53/p21 protein expression after transfection of HL-60 cells with vectors harboring A-like and non-A like mutations, in comparison with the expression of NPM1wt cells. As shown in Figure 3A, we observed a moderate reduction in p14ARF levels 8 h post transfection in the transfected cells (reduction of 11% and 26% in NPM1mutB and NPM1mut7, respectively) and marked reduction in the downstream pathway p53 and p21 proteins expression. Moreover, comparing the different mutations, we observed a greater reduction of p53 and p21 in cells transfected with A-like NPM1mutB (reduction of 51% and 73% respectively; Figure 3A) than in cells transfected with non-A-like NPM1mut7 (reduction of 11% and 31%, respectively, Figure 3A). To validate these results, experiments were repeated on a second cell line, SHI-1. In SHI-1, we confirmed that non-A-like NPM1mut7 maintained higher p53 and p21 levels than A-like NPM1mutB (Figure 3B), supporting our hypothesis that the residual NPM1 remaining in the nucleolus maintains p14ARF/p53/p21 pathway pro-apoptotic activity.

**Figure 4:**
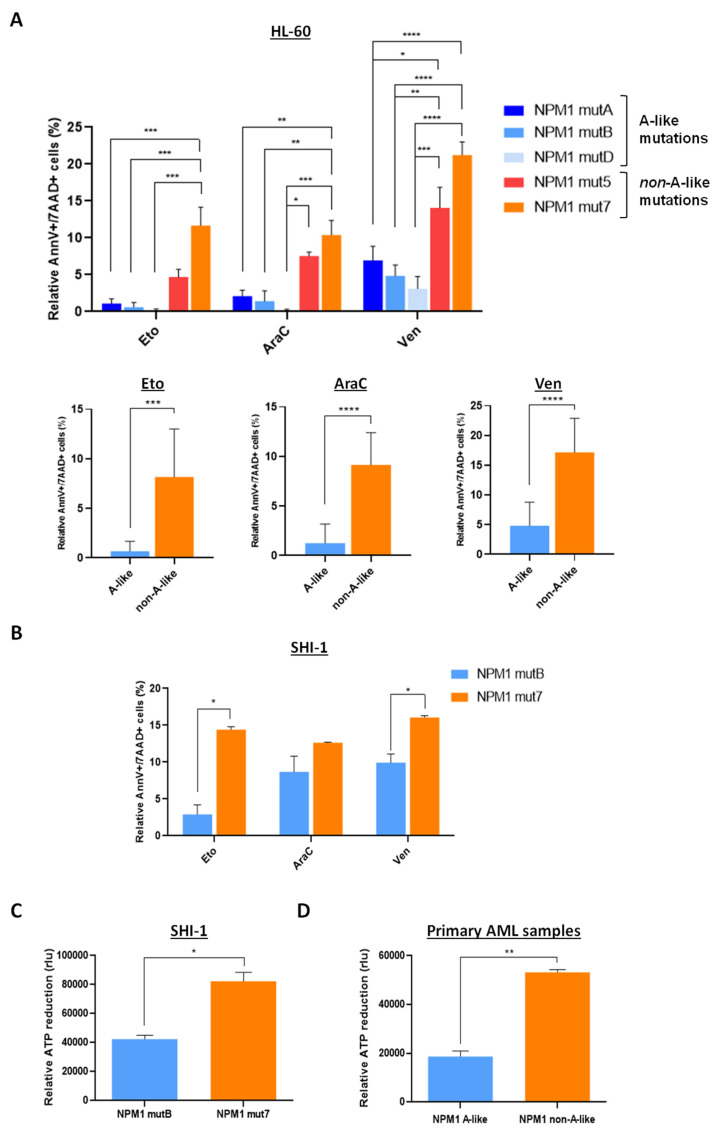
Treatment sensitivity in different NPM1-mutated cells. (**A**) Percentage of Annexin-V/7AAD positive cells, relative to DMSO, 48 h after Eto (1 µM), AraC (2.5 µM), Ven (5 nM), in HL-60 cells transfected with pEGFP-NPM1mutA, mutB, mutD (A-like) or pEGFP-NPM1mut5, mut7 (non-A-like) plasmids, gating on EGFP positive cells. In the lower panel is reported the mean of Annexin-V/7AAD positive cells after treatment, considering A-like and non-A-like mutations together. * *p*-value < 0.05, ** *p*-value < 0.01, *** *p*-value < 0.001, **** *p*-value < 0.0001. (**B**) Percentage of Annexin-V/7AAD positive cells in SHI-1 transfected with pEGFP-NPM1mutB (A-like) or pEGFP-NPM1mut7 (non-A-like) plasmids, gating on EGFP positive cells and relative to DMSO, after 48-h of treatment with Eto (1 µM), AraC (2.5 µM), or Ven (1 µM). * *p*-value < 0.05. (**C**) Reduction of cell viability measured by ATP assay after 24 h of treatment with Eto (1 µM), relative to DMSO, in SHI-1 cells transfected with pEGFP-NPM1mutB (A-like) or pEGFP-NPM1mut7 (non-A-like) vectors. * *p*-value < 0.05. (**D**) Reduction of cell viability measured by ATP assay after 24-h treatment with AraC (2.5 µM), relative to DMSO, in primary AML samples harboring A-like or non-A-like NPM1 mutations. ** *p*-value < 0.01. rlu: relative luminescence unit.

Based on these results, we next evaluated the impact of NPM1 different mutation types on the activation of apoptosis after drug exposure. We therefore treated cells for 48 h with Etoposide (Eto), Cytarabine (AraC), or with the BCL-2-targeting drug Venetoclax (Ven). As shown in Figure 4A, HL-60 cells expressing NPM1 mutations had a profound impairment of the activation of apoptosis, with NPM1 non-A-like mutations inducing a higher cell death after treatment as compared to A-like mutations (Figure 4A upper panel: apoptotic and dead cells after Eto treatment relative to DMSO: 1.1%, 0.62%, 0.17%, 4.7% and 11.67%; after AraC treatment relative to DMSO: 2.12%, 1.41%, 0.16%, 7.53% and 10.38%; after Ven treatment relative to DMSO: 6.94%, 4.85%, 3.11%, 14.07% and 21.23%; in NPM1mutA-, mutB-, mutD-, mut5- and mut7-cells, respectively; * *p* < 0.05, ** *p* < 0.01, *** *p* < 0.001, **** *p* < 0.0001). These results are even more evident when drug-induced apoptosis was analyzed clustering together the NPM1 A-like mutations compared with the non-A-like mutations (Figure 4A lower panel: average of apoptotic and dead cells of all the A-like versus non-A-like constructs pooled together: Eto treatment relative to DMSO: 0.68% in A-like vs 8.18% in non-A-like cells; after AraC treatment relative to DMSO: 1.25% in A-like vs 9.16% in non-A-like cells; after Ven treatment relative to DMSO: 4.84% in A-like vs 17.14% in non-A-like cells; *** *p* < 0.001, **** *p* < 0.0001). We confirmed the observed heterogeneity of NPM1 mutations in triggering apoptosis by treating another cell line, the SHI-1 cells with Eto, AraC, or Ven (Figure 4B, apoptotic and dead cells 48 h after Eto treatment, relative to DMSO: 2.9% and 14.4%; after AraC treatment relative to DMSO: 8.7% and 12.6%; after Ven treatment relative to DMSO: 9.9% and 16.1%, in cells transfected with NPM1mutB and NPM1mut7, respectively; * *p* < 0.05). Similar results were obtained by evaluating cell viability through ATP measurement 24 h after treatment with Eto: in SHI-1 transfected with NPM1mut7, treatment resulted in a significantly higher reduction of cell viability than in cells transfected with NPM1mutB (Figure 4C, * *p* < 0.05). To further validate these findings, we tested AraC in two NPM1-mutated AML primary samples, confirming that the non-A-like NPM1-mutated AML cells were more sensitive to the effect displayed by AraC than A-like-mutated cells (Figure 4D, ** *p* < 0.01). Moreover, we attempted to treat transfected cells with a combined treatment showing that this combo was more effective in NPM1mut7, but further drug combinations need to be tested in this context of NPM1-mutational status (Supplemental Figure S4).

## 4. Discussion

In patients affected by AML, NPM1-isolated mutations are markers associated with relatively good prognosis and, therefore, patients carrying these abnormalities are included in the standard risk prognostic group in national and international trials [32]. However, a wide NPM1 mutations spectrum has been described, with different frequency between adults and children [33]. A common biologic characteristic induced by NPM1 mutations is the relocation of the mutated protein to the cytoplasm, which is essential for leukemic transformation. In fact, while the N-terminal region constitutes the core domain mediating NPM1 oligomerization and interaction with other proteins, the C-terminus, where almost the totality of variants occurs, serves for nucleolar localization. The significance and effects of the different mutations have been only partially investigated and contrasting results were reported [25,26,34]. In the present work, we characterized the effect of NPM1 mutations, comparing the loss of either one or both Trp in NoLS domain (namely A-like and non-A-like mutations), by using different NPM1 mutant vectors.

We showed that in non-A-like NPM1 mutations, the residual Trp allowed a partial nucleolar NPM1 protein expression, partially sustaining its physiological function. An important feature of NPM1-mutated AML is the upregulation of HOX family genes [31], that we confirmed in our analysis. Even if the mechanism inducing the upregulation of HOX genes in NPM1-mutated patients remains unclear, it has been indubitably proved that it depends directly on the aberrant cytoplasmic localization of NPM1, since nuclear re-localization of the mutated protein induces the immediate downregulation of the HOX genes [35]. Our gene expression analysis showed that non-A-like NPM1-mutated AML cluster together and apart from the A-like ones. Of note, the two cases with A-like mutations did not cluster in the same dendrogram branch, probably due to the presence of FLT3ITD additional mutation in one of the A-like patients. Based on these observations, we then evaluated in vitro the expression of HOX genes in A-like or non-A-like-mutated AML cells, documenting a different expression of the most relevant HOXA5, HOXB5, and HOXA10 genes. These in vitro data suggest that the NPM1 mutational status has a potential different contribution on the aggressiveness of AML cells, since the over-expression of HOX genes was previously described to be correlated with poor survival of NPM1-mutated AML patients [31].

Another important feature associated with the NPM1 localization is represented by p53-dependent apoptosis. Here, we showed that p53 and p21 levels are modulated according to the number of Trp residues substituted at the C-terminus of NPM1. In particular, both p53 and p21 were less expressed in A-like-mutated cells than in non-A-like-mutated blasts, due to p14ARF reduction and cytoplasmic dislocation driven by A-like NPM1 mutation [5]. The decrease of p14ARF/p53/p21 proteins results in a lower induction of apoptosis in response to drug treatment. Conversely, in non-A-like NPM1-mutated cells, p53 still partially exerted its pro-apoptotic function, thus sustaining the drug response. According to this interpretation, we suggest that AML with a non-A-like mutation of NPM1 might be more sensitive to drugs than A-like NPM1-mutated AML. The latter could therefore preferentially benefit of new strategies of treatment, able to contrast the abnormal nuclear export of NPM1 mutant, for example by targeting either XPO1 [23] with the novel agent Eltanexor [36], or HOX hyper-expression by inhibiting MENIN-MLL interaction, with novel inhibitors such as MI3454 and VTP-50469 [37,38]. These approaches already showed a promising antileukemic activity against NPM1-mutated AML in mice [39].

Overall, our results prove that different NPM1 mutations mediate diverse clinical-biological characteristics of AML cells, suggesting that patients with AML harboring a NPM1 mutation may benefit from a refined sub-classification (A-like or non-A-like-mutated) and a subsequent tailored management including the possibility of novel target treatment strategies to improve their outcome.

## 5. Conclusions

This study suggests that the classification of NPM1-mutated AML in A-like or non-A-like cases can reflect different biological and clinical peculiarities. Further deconvolution of the heterogeneity of NPM1 mutations in pediatric AML may pave the way for more tailored and possibly effective therapeutic strategies.

## Figures and Tables

**Table 1:** Primers list.

Primers	Sequence
NPM1_mutA_F	5′ATCTCTGTCTGGCAGTGGAGGAAGTCTCTTTAAGAAAATAGTT 3′
NPM1_mutA_R	5′ACTGCCAGACAGAGATCTTGAATAGCCTCTTGGTCAGT 3′
NPM1_mutB_F	5′ATCTCTGCATGGCAGTGGAGGAAGTCTCTTTAAGAAAATAGTT 3′
NPM1_mutB_R	5′ACTGCCATGCAGAGATCTTGAATAGCCTCTTGGTCAGT 3′
NPM1_mutD_F	5′ATCTCTGCCTGGCAGTGGAGGAAGTCTCTTTAAGAAAATAGTT 3′
NPM1_mutD_R	5′ACTGCCAGGCAGAGATCTTGAATAGCCTCTTGGTCAGT 3′
NPM1_mut5_F	5′TGGCAGAGAATGGAGGAAGTCTCTTTAAGAAAATAGTTTAA 3′
NPM1_mut5_R	5′CTCCATTCTCTGCCAGAGATCTTGAATAGCCTCTTG 3′
NPM1_mut7_F	5′CAGTGCTTTTCAAAAGTCTCTTTAAGAAAATAGTTTAAACAA 3′
NPM1_mut7_R	5′ACTTTTGAAAAGCACTGCCAGAGATCTTGAATAGCCT 3′

## Data Availability

The data presented in this study are openly available in GEO DataSets, reference number GSE75461.

## Supplementary Materials

The following are available online at https://www.mdpi.com/article/10.3390/cancers13143457/s1. Figure S1: Physiological roles of NPM1, Figure S2: HOXA and HOXB gene expression according to mutational status, Figure S3: HOXA gene expression according to different NPM1 mutations, Figure S4: Treatment sensitivity according to different NPM1 mutations, Figure S5: Whole western blot images.
