# Supplementary material for: NPM1 Mutational Status Underlines Different Biological Features in Pediatric AML
Source: Cancers (Basel). 2021 Jul 10;13(14):3457. doi: 10.3390/cancers13143457 (PMC8304368; doi:10.3390/cancers13143457)
Supplement: Supplementary file 1 [file cancers-13-03457-s001.zip › cancers-1282457-supplementary.pdf]

cancers

Supplementary Materials

NPM1 Mutational Status Underlines Different Biological Features in Pediatric AML

Claudia Tregnago, Maddalena Benetton, Davide Padrin, Katia Polato, Giulia Borella, Ambra Da Ros, Anna Marchetti, Elena Porcù, Francesca Del Bufalo, Cristina Mecucci, Franco Locatelli and Martina Pigazzi

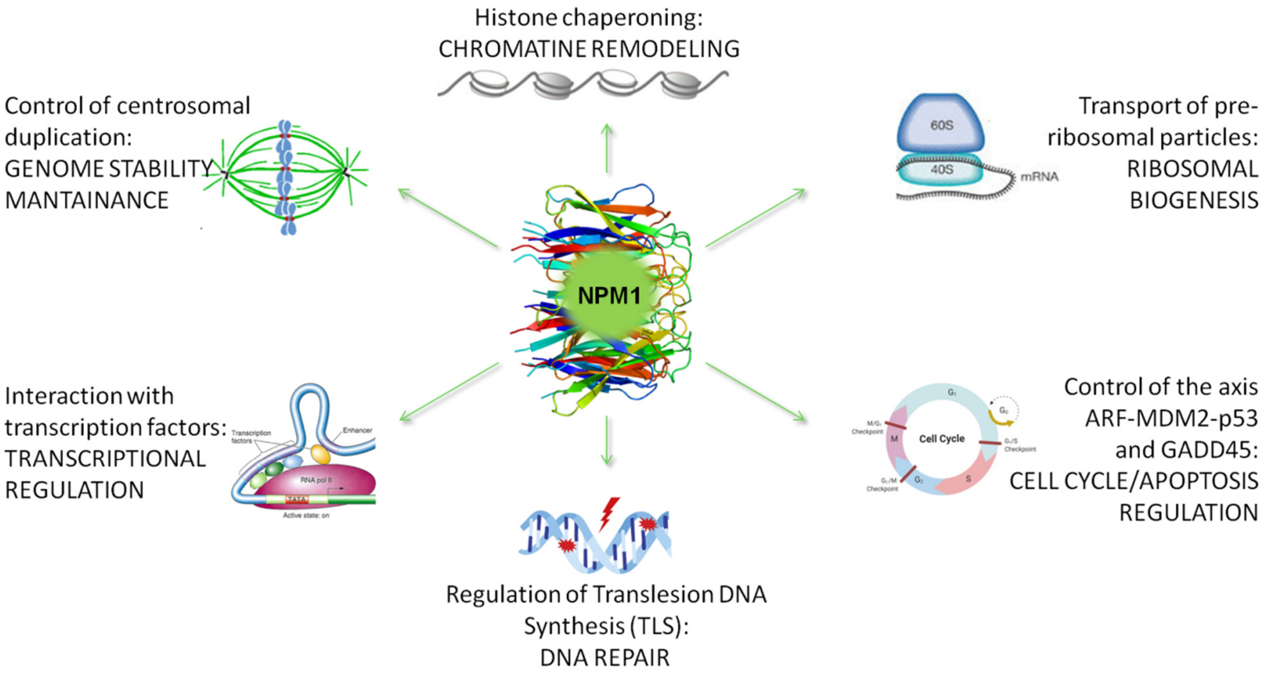

Figure S1. Physiological roles of NPM1. Main physiological functions exerted by NPM1wt contributing to the maintenance of cell homeostasis.

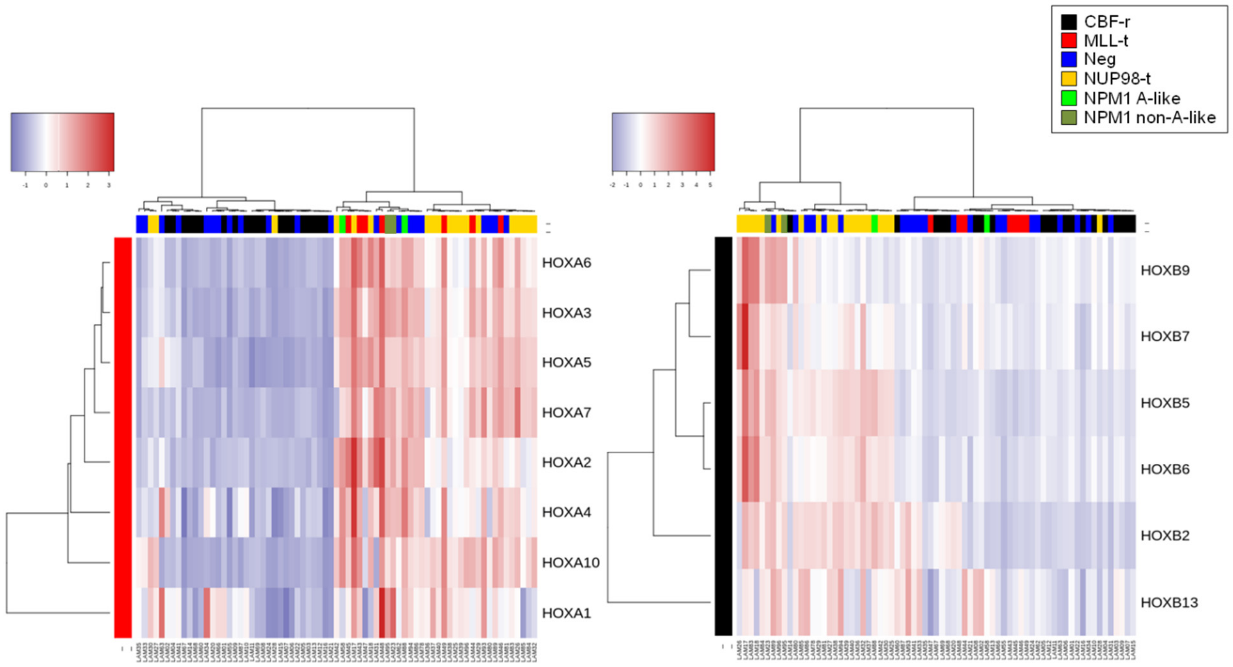

Figure S2. HOXA and HOXB gene expression according to mutational status. Hierarchical clustering analyses of 71 AML patients for HOXA (left) and HOXB (right) genes. AML genetic markers are reported in the legend.

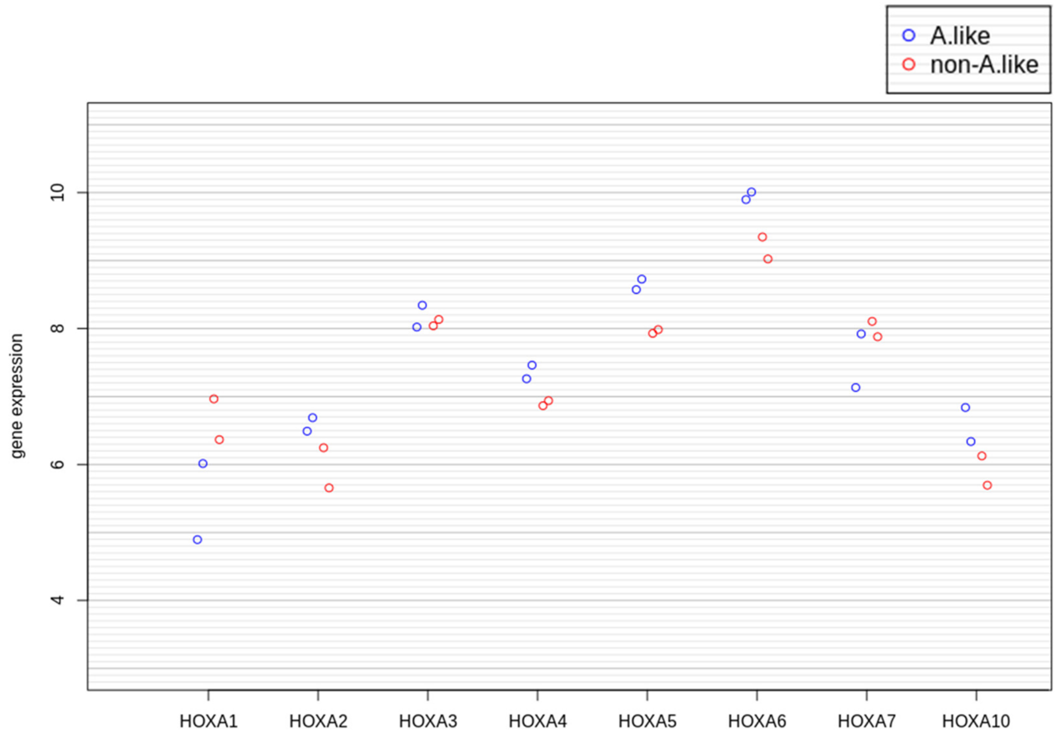

Figure S3. HOXA gene expression according to different NPM1 mutations. Dot plot showing the expression of HOXA genes in the AML samples harboring A-like *NPM1* mutations (blue dots, n=2) and the AML with non-A-like *NPM1* mutations (red dots, n=2) in the GSE75461 cohort.

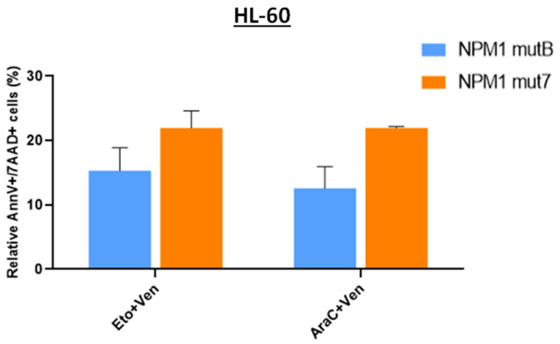

Figure S4. Treatment sensitivity according to different NPM1 mutations. Percentage of Annexin-V/7AAD positive cells, relative to DMSO, 48 hours after combo treatments of Eto (1 μM) or AraC (2.5 μM) with Ven (5 nM), in HL-60 cells transfected with pEGFP-NPM1mutB or pEGFP-NPM1mut7.

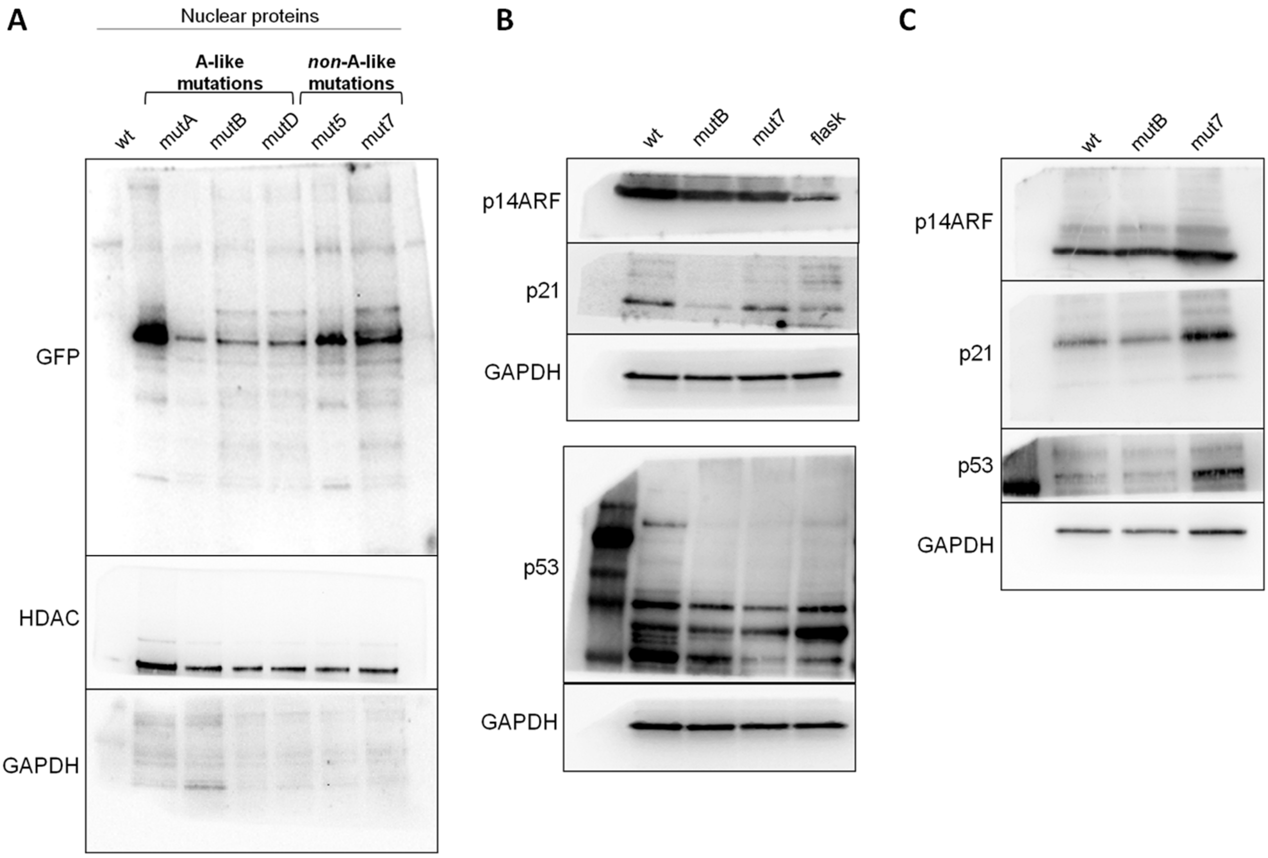

Figure S5. Whole western blot images. The whole western blot images of the blots presented in Figure 1B (A), 3A (B) and 3B (C).
